# Clustered DNA Lesions Containing 5-Formyluracil and AP Site: Repair via the BER System

**DOI:** 10.1371/journal.pone.0068576

**Published:** 2013-08-06

**Authors:** Ekaterina A. Belousova, Inna A. Vasil'eva, Nina A. Moor, Timofey S. Zatsepin, Tatiana S. Oretskaya, Olga I. Lavrik

**Affiliations:** 1 Laboratory of Bioorganic chemistry of Enzymes, Institute of Chemical Biology and Fundamental Medicine, Novosibirsk, Russia; 2 Chemistry Department of Moscow State University and A.N. Belozersky Institute of Physico-Chemical Biology, Moscow, Russia; New England Biolabs, Inc., United States of America

## Abstract

Lesions in the DNA arise under ionizing irradiation conditions or various chemical oxidants as a single damage or as part of a multiply damaged site within 1–2 helical turns (clustered lesion). Here, we explored the repair opportunity of the apurinic/apyrimidinic site (AP site) composed of the clustered lesion with 5-formyluracil (5-foU) by the base excision repair (BER) proteins. We found, that if the AP site is shifted relative to the 5-foU of the opposite strand, it could be repaired primarily via the short-patch BER pathway. In this case, the cleavage efficiency of the AP site-containing DNA strand catalyzed by human apurinic/apyrimidinic endonuclease 1 (hAPE1) decreased under AP site excursion to the 3'-side relative to the lesion in the other DNA strand. DNA synthesis catalyzed by DNA polymerase lambda was more accurate in comparison to the one catalyzed by DNA polymerase beta. If the AP site was located exactly opposite 5-foU it was expected to switch the repair to the long-patch BER pathway. In this situation, human processivity factor hPCNA stimulates the process.

## Introduction

During the cell cycle, the DNA bases are modified by different endogenous factors in the cellular milieu. Moreover, unfavorable exogenous physicochemical factors (such as ionizing irradiation, UVA- and UVB-rays, cigarette smoke, products of incomplete combustion fuel in the atmosphere or drug treatment) are important boosters of DNA damage. The action of ionizing irradiation or active/radical forms of oxygen leads to the formation of multiple DNA lesions. Lesions located in the range of one or two turns of the DNA helix are designated multiply damaged sites or clusters. Using computer simulation algorithm it was calculated that the action of high linear energy transfer irradiation could result in cluster formation containing 10–25 individual damages to 100–200 base pairs [1]. Tandem lesions located in one or two DNA strands potentially enhance the risk of the appearance of mutations, since cluster may include a few types of lesions that can be removed from the DNA by different repair systems depending on their nature.

One of the major lesions arising in the cell as a product of the thymine methyl group under UVA- or ionizing irradiation is 5-formyluracil (5-foU) [2]. The presence of 5-foU in the DNA does not lead to replication block on both DNA strands; however high-fidelity DNA polymerases such as the Klenow fragment of DNA polymerase I, DNA polymerase alpha and gamma are able to incorporate any of the 4 dNTPs opposite 5-foU allowing transition and transversions [3]. The 5-foU residue is weakly mutagenic, with mutation frequencies in double-stranded vectors in the range of 0.01–0.04% [4]. One of the troublesome outcomes of the existence of 5-foU is the formyl group. First, it can interact with its surroundings, for example with the amino group of DNA binding proteins, and form covalent adducts via Schiff bases [5]. Second, the N-glycosylic bond of 5-formyluridine is less stable than that of 2'-deoxyuridine even in physiological conditions, which results in the generation of an apurinic/apyrimidinic site (AP site) in the DNA chain that has no coding base, and goes to spontaneous or enzymatic cleaving single-strand break [6]. Additionally, the presence of AP site in DNA strand force the linear form of DNA to kink which may lead to changing the recognition of the damaged site by repair proteins [7]. Thus, if 5-foU in the DNA is located close to the AP site or its precursor thereby forming a cluster, it can lead to additional danger relative to the formation of single- or double-strand breaks or involving an alternative repair system.

Data exist concerning 5-foU repair in reconstituted systems and also in cell-free extracts from HeLa and MRC5 cells [8], [9]. It was suggested that basically 5-foU is repaired by the base excision repair system (BER). Several DNA glycosylases from *Escherichia coli* and human cells are known that can remove this lesion from DNA with different efficiencies [10], [11]. However, some facts demonstrate that 5-foU can be removed by the nucleotide excision repair system (NER). The group of Hanaoka observed that one of the main initiators of the NER process, XPC-HR23B, interacts with DNA duplexes containing all four base pairs coupled to 5-foU [12]. Furthermore, the region of the DNA with 5-foU is effectively excised from such DNA duplexes by cell-free NER extracts, whose efficiency depends on the instability of the base pair but in this case the point mutation is fixed. Another NER factor RPA interacts with ssDNA containing 5-foU [13].

Thus, in the situation when 5-foU and the AP site compose one cluster, the cell must coordinate the choosing of the correct repair system (BER or NER) as well as the order of lesion repair. In any case, the appropriate DNA polymerase can provide DNA synthesis using the damaged template. If this synthesis is proceeds in a frame of the BER process, the role of such DNA polymerase can play DNA polymerase beta or lambda [14].

In this study, we explored the repair opportunity of the AP site composed of the clustered lesion by the BER proteins. As DNA substrates, DNA duplexes were used containing 5-foU and uracil as AP site precursor in the different strands displaced from each other towards the 3'- or 5'-side, i.e. in the positive or negative orientation (Table 1). The lesions are said to be in the positive orientation when the opposing strand lesion is 3′- of the base opposite the reference lesion, and to be in the negative orientation when the lesion in the opposite strand is 5′- of the base opposite the reference lesion. The main repair activities investigated were the activity of human apurinic/apyrimidinic endonuclease 1 (hAPE1), DNA polymerase activity of DNA polymerases beta and lambda and their cooperation with endonuclease activity of human flap endonuclease 1 (hFEN1). Additionally, the influence of the human X-ray crosscomplementing protein 1 (hXRCC1) and processivity factor hPCNA on the BER process was studied.

**Table 1:** DNA substrate constructs used in the present study.

name	sequences	primer length (nts)^a^
DNA1	5'-GACTACATTTCATCTGGCTTGGGCTTCATCGTTGTC**dU^f^**CAGACCTGGTGGATACCG	17
	3'-CTGATGTAAAGTAGACCGAACCCGAAGTAGCAACAG A **O**TCTGGACCACCTATGGC*	
DNA2	5'-GACTACATTTCATCTGGCTTGGGCTTCATCGTTGTC**dU^f^**CAGACCTGGTGGATACCG	18
	3'-CTGATGTAAAGTAGACCGAACCCGAAGTAGCAACAG **O** GTCTGGACCACCTATGGC*	
DNA3	5'-GACTACATTTCATCTGGCTTGGGCTTCATCGTTGTCd**U^f^**CAGACCTGGTGGATACCG	19
	3'-CTGATGTAAAGTAGACCGAACCCGAAGTAGCAACA**O** A GTCTGGACCACCTATGGC*	
DNA4	5'-GACTACATTTCATCTGGCTTGGGCTTCATCGTTGTC**T**CAGACCTGGTGGATACCG	18
	3'-CTGATGTAAAGTAGACCGAACCCGAAGTAGCAACAG**O**GTCTGGACCACCTATGGC*	

a primer length – the length of the primed oligonucleotide created under hAPE1 activity, **dU^f^** –5-formyl-2'-deoxyuridine, **O** – AP site, * –^32^P-radioactive label.

## Materials and Methods

### Materials

Synthetic oligodeoxyribonucleotides were obtained from GenSet (Switzerland). Reagents for electrophoresis and the basic components of buffers were from ®Sigma (USA). (γ-^32^P)ATP (specific activity 5000 Ci/mmol) was from the Laboratory of Biotechnology (Institute of Chemical Biology and Fundamental Medicine, Novosibirsk, Russia). T4 polynucleotide kinase and T4 DNA ligase were from ®Biosan (Novosibirsk, Russia). Ultra pure dNTPs were from ®Promega (USA).

### Proteins

Human recombinant DNA pol lambda and DNA pol beta were purified from *E.coli* BL21(DE3) RP cells as described in [15] and [16], respectively. hPCNA was purified according to [17]. Uracil-DNA glycosylase (UDG) was from *E.coli*, and hAPE1 and hFEN1 were purified and kindly provided by Svetlana Khodyreva (Institute of Chemical Biology and Fundamental Medicine Siberian Branch of the RAS, Novosibirsk, Russia).

### XRCC1 purification

Human full-length XRCC1with an N-terminal histidine tag was expressed in *E.coli* from the plasmid pET16BHX kindly supplied by J.P.Radicella (from Dr Keith W. Caldecott, University of Sussex, Brighton, UK) and purified as described in [18] with some modifications. In particular, *E.coli* BL21(DE3) Rossetta cells containing the plasmid were grown in LB medium to OD_600_ 0.4 and induced with 0.5 mM IPTG for 3 h. After centrifugation, the cells were quick-frozen at −40°C. The pellets were thawed on ice and resuspended in 20 ml ice-cold sonication buffer containing 50 mM Na phosphate, pH 7.8, 300 mM NaCl, 1% NP-40, 1 mM β-mercaptoethanol (β-Me), 10% glycerol, 1 mM PMSF and 1 mM benzamidine (Bz), and sonicated on ice (10×15 s with 100 s cooling intervals). Cellular debris was removed by centrifugation (18000***g***, 45 min, 4°C). The protein was further purified on a Ni-sepharose fast flow His-tag affinity column (GE Healthcare, Sweden). Imidazole, pH 7.2 was added to the supernatant (23 ml) to 5 mM final concentration, and the mixture was loaded onto 3 ml Ni-resin and stirred on ice for 1 h. The flow-through was then collected. The column was washed with 3 column volumes (CV) of each buffer A (50 mM Na phosphate, pH 7.2, 300 mM NaCl, 0,1% NP-40, 3.5 mM β-Me, 1 mM PMSF and 1 mM Bz), containing 5, 40 and 50 mM imidazole and 10 ml fractions collected. The column was then washed with 3 CV of each buffer A, containing 200 and 300 mM imidazole, with 1.5 ml fractions collected. Fractions containing XRCC1 were pooled and diluted with buffer B (50 mM Na phosphate, pH 7.2, 25 mM NaCl, 0.05% NP-40, 10 mM β-Me, 5% glycerol, 0.5 mM EDTA, 1 mM PMSF and 1 mM Bz) to 80 mM final NaCl concentration, and loaded onto a 5 ml Heparine-sepharose 6 Fast Flow column (®GE Healthcare, Sweden). The protein was eluted with a 70 ml NaCl linear gradient (80–700 mM) in buffer C (50 mM Na phosphate, pH 7.2, 10 mM β-Me, 5% glycerol, 0.5 mM EDTA). XRCC1-containing fractions which were determined by acrylamide gels stained with Coomassie blue R250 were concentrated on Vivaspin centrifugal concentrators with a PES membrane 10 kDa. The final purity was ≥80%, as estimated by acrylamide gels stained with Coomassie blue R250. To test the hXRCC1 activity, its ability to form complexes with hAPE1 and DNA polymerase beta was assayed according to [18] with minor changes. In particular, 350 pmol hXRCC1, 114 pmol hAPE1 and 130 pmol DNA polymerase beta in 40 μl reaction mixture were used for the analysis. Buffer A contained 1 mM β-Me instead of DTT, and incubation time was increased to 1 h. Before electrophoresis all mixtures were incubated with an extra 5 mM DTT on ice overnight. It was observed that the recombinant protein is able to perform protein-protein interactions with hAPE1 and DNA polymerase beta (data presented in Fig. S1).

### Oligonucleotide substrates

All modified oligonucleotides were synthesized by the phosphoramidite method on an automatic ABI 3400 DNA synthesizer (®Applied Biosystems, USA) using the conditions recommended by the manufacturer, and double purified a) by electrophoresis in a 20% polyacrylamide/7 M urea gel and b) by RP-HPLC with an acetonitrile gradient in 0.1 M ammonium acetate (pH 7). HPLC purification was carried out on AKTA Purifier equipped with Jupiter C18 or C5 column (®Phenomenex, size 4.6×250 mm, 5 μm), at 45°C, 1 ml/min with a UV-Vis detector. T, dA(Bz), dG(iBu), dC(Ac), 5-(1,2-diacetoxyethyl)-2'-deoxyuridine phosphoramidites and Universal UnyLinker CPG Support 500 Å were purchased from ChemGenes (USA). To generate the formyl group, 300 pmol of oligonucleotide were incubated with 500 nmol fresh NaIO_4_ and 11.5 μmol NaOAc, pH 4.5, in 50 μl the reaction mixture at room temperature for 1 h [19]. The reaction was terminated by adding 70 μl of 3 M LiClO_4_ and pure acetone to 1.5 ml. The oligonucleotide precipitate was generated at −40°C for 2 h, centrifuged, washed twice with 1 ml cooled acetone (4°C), air dried, dissolved in water to the required concentration and used for annealing.

### Preparation of 5′-[^32^P]labeled primers

Radioactive label was incorporated into the 5′-end of the U-containing oligonucleotide using phage T4 polynucleotide kinase as described [20]. The reaction mixture (10 μl) contained 0.5 μM primer, 10 MBq [γ-^32^P]ATP, and 5 U T4 polynucleotide kinase. The reaction was performed at 37°C for 30 min, and then further overnight at 4°C. Nucleotide material was resolved by 20% denatured PAGE, visualized by autoradiography, and isolated by electroelution onto DE81-paper (Whatmann) in 50 mM Tris-borate buffer, pH 8.3. The product was eluted from DE81-paper by five portions (20 μl per portion) of 3 M LiClO_4_, and then 1.2 ml cooled acetone (4°C) was added to the eluate. The probe was incubated at –40°C for 2 h. The precipitate was collected by centrifugation, air dried, dissolved in water to the required concentration and used for annealing.

### Annealing of DNA oligonucleotides

Two oligonucleotides in a 1∶1.3 molar ratio where annealed in water in which 1 is the 5'-[^32^P]phosphorylated U-containing oligonucleotide and 1.3 is the 5-foU/T-containing oligonucleotide. The solution was heated at 97°C for 5 min and slowly cooled to 73°C, then incubated at 73°C for 15 min and slowly cooled to room temperature. The amount of duplex DNA substrate was controlled by native 12% polyacrylamide gel electrophoresis (PAGE) analysis PAAG. The oligonucleotide substrates used are presented in the Table 1.

*AP-site* was obtained using the glycosylase activity of uracil-DNA glycosylase directly before the following reactions. Mixtures contained 1 pmol of 5'-[^32^P]phosphorylated DNA substrate and 0.1 U of UDG in 10 μl water were incubated at 37°C for 30 min and used for the following reactions. Complete excision of uracil was confirmed by PAGE analysis in each case (specified on the figures).

All the following reactions were carried out in the presence of 1 mM MgCl_2_ or MnCl_2_. This concentration of bivalent ions was shown to be optimal for the TLS activity (i.e. translesion synthesis) of DNA polymerases beta and lambda in the range of 0.1–10 mM for MgCl_2_ and 0.05–1.2 mM for MnCl_2_ (data not shown).

#### Endonuclease activity of hAPE

Endonuclease activity of hAPE1 was investigated in reaction buffer (TDB buffer) containing 50 mM Tris-HCl (pH 7.5), 0.5 mM ditiothreithol, 0.25 mg/mL bovine serum albumin, and 1 mM MgCl_2_ or MnCl_2_ as follows. First, the kinetics of the endonuclease reaction by APE1 was studied. For this, the reaction mixtures (final volume 80 μl) including 10 nM 5'-[^32^P]phosphorylated DNA substrate, 10 nM hAPE1 and 1 mM MgCl_2_ or MnCl_2_ in TDB buffer were incubated at 37°C for 20 sec, 40 sec 1, 1.5, 2, 3, 5, 10 and 15 min. The aliquots (10 μl) were collected at the indicated times, and the reaction products were stabilized at 4°C for 30 min after addition of NaBH_4_ to a concentration of 20 mM. The reactions were terminated by adding the gel loading solution (90% formamide, 0.1% xylene cyanol and 0.1% bromophenol blue) and heated at 97°C for 5 min. The mixtures were resolved on a 20% polyacrylamide gel containing 7 M urea in TBE buffer [21]. The gels were dried and subjected to autoradiography and/or phosphorimaging for quantitation using the Molecular Imager Pro FX+ and “Quantity One” software (®Bio-Rad, USA), and analyzed using OriginPro7.5 (®Microcal Software, USA). Based on the results obtained, a 1.5 min interval was chosen as the reaction time. Second, the DNA cleavage efficiency by APE1 was estimated. All mixtures (final volume 10 μl) included 10 nM 5'-[^32^P]phosphorylated DNA substrate, 0.1–10 nM hAPE1 and 1 mM MgCl_2_ or MnCl_2_ in TDB buffer. All the reaction mixtures were incubated at 37°C for 1.5 min. The reaction products were stabilized by addition of NaBH_4_ to 20 mM at 4°C for 30 min. The reactions were terminated and analyzed as outlined above. To estimate the kinetic parameters K_m_ and V_max_, where the V_max_ value was measured as the amount of cleavage product in pmol generated per sec, and the K_m_ value was the DNA concentration needed to obtain a rate equal to V_max_/2, the hyperbolic equation was used. The influence of hXRCC1 on the endonuclease activity of hAPE1 was studied as follows. All mixtures (final volume 10 μl) included 10 nM 5'-[^32^P]phosphorylated DNA substrate, 1 nM hAPE1 and 0.1–500 nM hXRCC1 and were incubated at 37°C for 1.5 min. The reaction products were stabilized by addition of NaBH_4_ to 20 mM at 4°C for 30 min. The reactions were terminated and analyzed as outlined above. All experiments were repeated several times.

### DNA pol assay

DNA synthesis catalyzed by DNA polymerases beta and lambda was performed in TDB buffer. First, all DNA substrates were treated by UDG (as mentioned above). Then all mixtures (final volume 10 μl) included 10 nM 5'-[^32^P]phosphorylated DNA substrate treated by UDG, 2 nM hAPE1 and 1 mM MgCl_2_ or MnCl_2_ in TDB buffer were incubated at 37°C for 5 min to complete cleavage of the AP site-containing DNA strand which was confirmed by PAGE analysis in all cases (specified in the figures). DNA polymerase beta or lambda (10 nM), and 50 μM dNTP (dGMP using DNA1, 3 and dAMP using DNA2, 4) at the reconstitution of the short-patch BER pathway, or 50 μM dNTPs and 50 nM hFEN1 at the reconstitution of the long-patch BER pathway were then added. All reactions were additionally incubated at 37°C for 15 min, then terminated and analyzed as outlined above. All experiments were reproduced several times.

The influence of hXRCC1 on the incorporation of dNMP was studied as follows. All mixtures (final volume 10 μl) included 10 nM 5'-[^32^P]phosphorylated DNA substrate treated with UDG and APE1, 10 nM DNA polymerase beta or lambda, 1 mM MgCl_2_ or MnCl_2_, 50 μM dNTP (dGMP using DNA1, 3 and dAMP using DNA2, 4), and 0.1–500 nM hXRCC1. All reactions were incubated at 37°C for 15 min, then terminated and analyzed as outlined above. All experiments were reproduced several times.

#### Michaelis constants, Km, and maximum velocities, Vmax, of DNA synthesis catalyzed by DNA pols beta or lambda

Michaelis constants, Km, and maximum velocities, Vmax, of DNA synthesis catalyzed by DNA pols beta or lambda were determined in two steps. In the first step, the kinetics of dNMP incorporation into different DNA substrates by the DNA polymerases beta and lambda were estimated. The reaction was performed in 90 μl of a mixture containing 10 nM DNA polymerases beta or lambda, 50 μM dNTP, 10 nM 5'-[^32^P]phosphorylated DNA substrate treated with UDG and hAPE1 (as mentioned above), and 1 mM MgCl_2_ or MnCl_2_ in TDB buffer at 37°C. Aliquots (10 μl) were removed after selected times in the range of 0–40 min. The reaction was terminated by adding the gel loading solution. The reaction products were separated as described above. In the next step the Michaelis constants, *K*_m_, and the maximum velocities, V_max_, of DNA synthesis catalyzed by DNA polymerase beta or lambda were determined by the variation of dNTP concentration from 0.5 to 50 μM of each dNTP, and the reaction products were analyzed as described above. The data were fitted according to the Michaelis–Menten kinetic equation [22]. The k_cat_ values were calculated as V_max_/e_0_, where e_0_ is the total concentration of DNA polymerase in the reaction mixture.

#### The effect of hPCNA on the strand-displacement activity of DNA pols beta and lambda

The effect of hPCNA on the strand-displacement activity of DNA pols beta and lambda was studied as follows. The reaction mixtures (final volume 10 μl) contained 10 nM 5'-[^32^P]labeled DNA substrates treated with UDG and hAPE1 (as mentioned above), 10 nM DNA polymerase beta or lambda, 5 μM each dNTP, 50 μM hFEN1, 50 or 100 nM hPCNA, and 1 mM MgCl_2_ or MnCl_2_ in TDB buffer. All reactions were incubated at 37°C for 15 min. The reaction products were analyzed as outlined above. All experiments were reproduced several times.

#### AP site repair via the short- and long-patch BER pathways

AP site repair via the short- and long-patch BER pathways was carried out as defined for the study of the DNA polymerase assay. Aliquots were removed before adding each protein. At the final step, 1 U of T4 DNA ligase was added to 10 μl reaction mixture and incubated at 20°C for 20 min. The reaction products were analyzed as outlined above. All experiments were reproduced several times.

## Results

### Endonuclease activity of hAPE1 on DNA substrates containing cluster lesions

**Figure 1:**
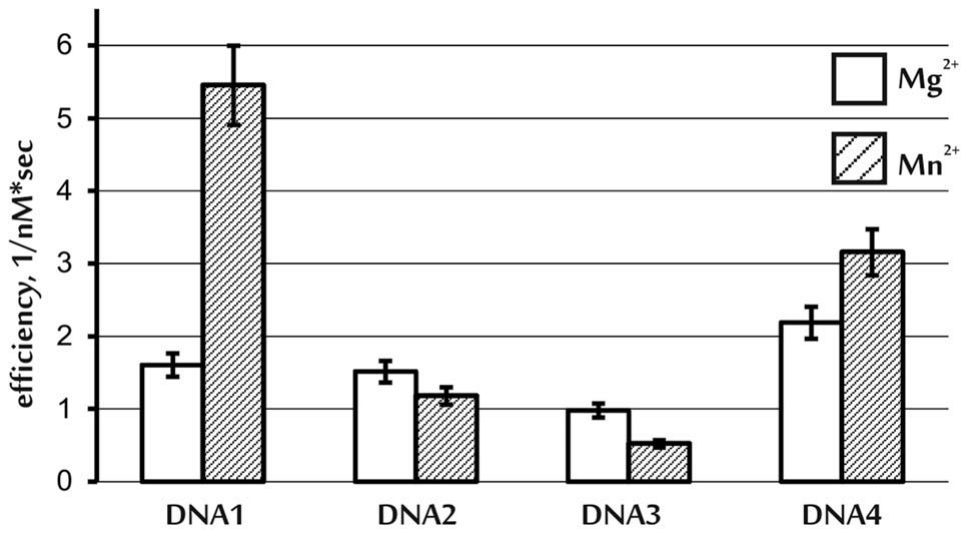
Efficiency (kcat/Km, 1/nM⋅sec) of the DNA endonuclease cleavage of the AP-DNA strand by hAPE1. Empty and shaded columns designate the values obtained in the presence of Mg^2+^ and Mn^2+^ ions, respectively. The results are presented as the average value of four independent experiments. Standard error was estimated as 10%.

The major enzyme that initiates the repair of AP sites during the BER process, is hAPE1 [23]. Here, we investigated the influence of the position of 5-foU relative to the AP site in the complementary DNA strand on the endonuclease activity of hAPE1 in the presence of Mg^2+^ or Mn^2+^ ions. To this end the kinetic parameters K_m_ and V_max_ were determined, and the efficiency of endonuclease cleavage of the AP-DNA strand catalyzed by hAPE1 calculated as the ratio of k_cat_ to K_m_ (Materials and Methods; Fig. 1). In the presence of Mg^2+^ ions the cleavage efficiency of the AP-containing DNA strand did not depend on the position of the 5-foU on the other strand. However, in the presence of Mn^2+^ ions the cleavage efficiency of AP-DNA strand decreased upon AP site excursion to the 3'-side relative to the lesion in the other DNA strand. It is noteworthy that in the last case, cleavage efficiency by hAPE1 was almost 2-fold higher using damaged substrate DNA1 rather than control DNA, DNA4. These results can be explained by the formation of a more productive intermediate complex including hAPE1, DNA and Me^2+^ and the simultaneous existence of the appropriate reciprocal orientation of the AP site and 5-foU:A pair in DNA1 in the presence of Mn^2+^ [24], [25].

### Influence of hXRCC1 on the endonuclease cleavage of AP-DNAs

**Figure 2:**
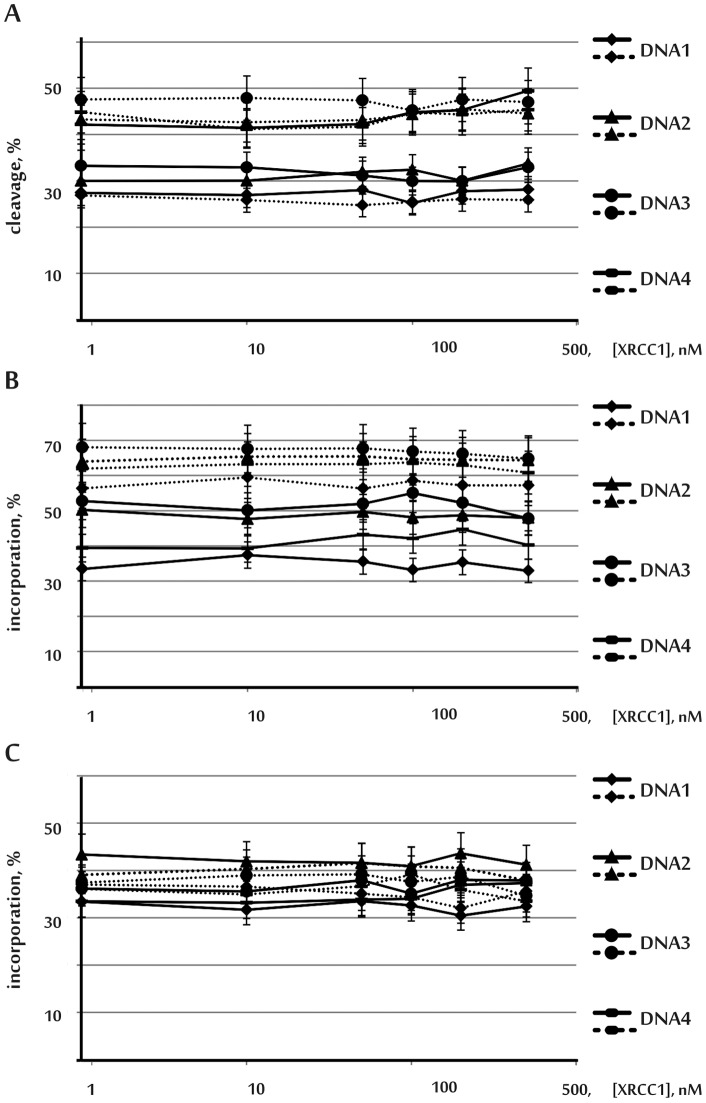
Influence of hXRCC1 on the endonuclease activity of hAPE1 (A), and on the incorporation of dNMP catalyzed by DNA polymerases beta (B) and lambda (C). The data are presented in logarithmic scale. Solid lines designate the yield of the reaction products obtained in the presence of Mg^2+^ ions; dotted lines designate the yield of the reaction products obtained in the presence of Mn^2+^ ions. The yield of the reaction products is the ratio of the reaction products over the initial amount of substrate expressed as a percentage. dGTP was used for the reactions with DNA1 and DNA3, and dATP – for the reactions with DNA2 and DNA4. The results are presented as the average value of four independent experiments. Standard error was estimated as 10%.

Many studies have suggested that XRCC1 plays a central role due to its action as coordinator of the BER process [24]. It is known, that hXRCC1 physically interacts with hAPE1 and DNA polymerase beta, and modulates their endonuclease and polymerase activity [26], [27]. Here, hXRCC1 was expressed in *E.coli*, and complex formation of the purified protein with recombinant hAPE1 and DNA polymerase beta was demonstrated (Fig. S1). The influence of hXRCC1 on the endonuclease activity of hAPE1 was investigated using AP-DNAs containing 5-formyl-2'-deoxyuridine (Fig. 2, A): addition of hXRCC1 up to 500 nM did not affect AP-DNA cleavage.

### dNMP incorporation catalyzed by DNA polymerases beta and lambda

**Figure 3:**
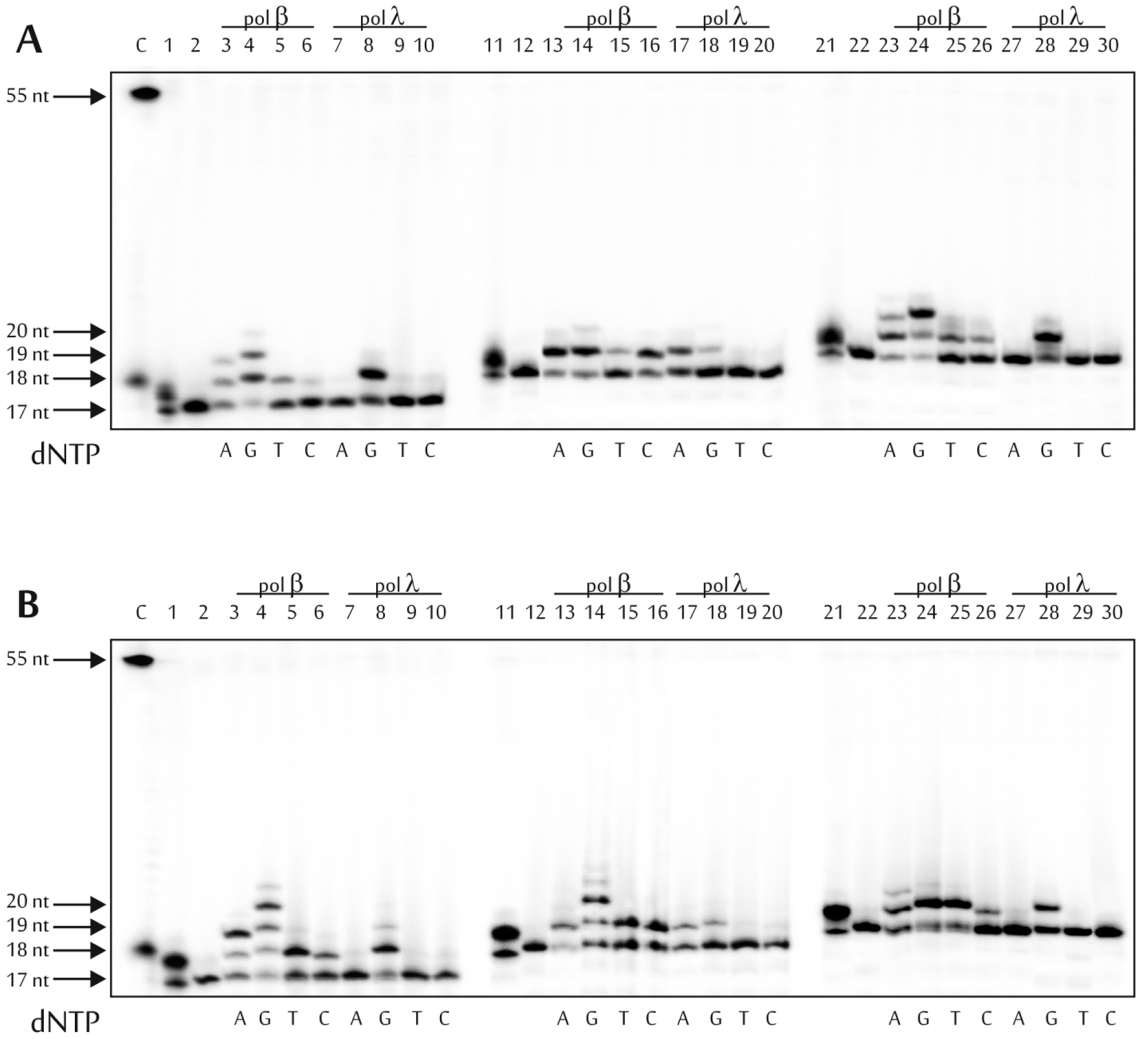
Specificity of dNMP incorporation catalyzed by DNA polymerases beta and lambda in the presence of Mg^2+^ (A) or Mn^2+^ (B) ions using DNA1 (lanes 1-10), DNA2 (lanes 11–20) and DNA3 (lanes 21–30). Lane C, position of the 5'–(^32^P)labeled 55 and 18 nt-long DNA; lanes 1, 11 and 21, initial DNA substrate after UDG treatment; lanes 2, 12 and 22, initial DNA substrate after sequential treatment by UDG and hAPE1; 18, 19 and 20 nt, lengths of the reaction products. The autoradiographs present the results from one of three independent experiments.

To simulate the short-patch BER pathway, dNMP incorporation catalyzed by DNA polymerase beta or lambda into 5-foU-containing DNAs exactly after AP-DNA cleavage by hAPE1was examined (Fig. 3). Clearly, DNA polymerase lambda is the more accurate enzyme during DNA synthesis across 5-foU. Independently from the reaction conditions, this enzyme could incorporate correct dNMP: dGMP as a complement to template C using DNA1 and DNA3, and dAMP as complement to the initial template T using DNA2 (Fig. 3). dGMP incorporation into the DNA strand from DNA2 in the presence of Mg^2+^ (∼10%) and Mn^2+^ (∼25%) ions could be explained, on one hand, by the stability of the 5-foU-G base pair, and on the other hand, by a template slippage mechanism as described for DNA polymerase lambda [28], [29]. The most effective and accurate DNA synthesis catalyzed by DNA polymerase lambda was observed using DNA1 and DNA3 (Table 2, and Table S1).

**Table 2:** Efficiency (k_cat_/K_m_, μM^−1^⋅sec^−1^) of dNMP incorporation by DNA polymerases beta and lambda via the short-patch BER pathway.

	pol λ		pol β	
	Mg^2+^	Mn^2+^	Mg^2+^	Mn^2+^
DNA1/dGMP	0.171	3.62	3.92	32.6
DNA2/dAMP	0.036	0.44	0.81	8.20
DNA3/dGMP	0.020	2.3	2.36	9.35
DNA4/dAMP	0.006	0.23	1.14	96.0

Note: the results are presented as the average value of three independent experiments. Standard error was estimated as 10%.

DNA polymerase beta is an extremely error-prone enzyme that can catalyze the elongation of the 3'-end of the primer opposite 5-foU and also close to the lesion using any dNMP (Fig. 3). Most likely the incorporation of dCMP and dTMP is associated with the low fidelity of DNA synthesis catalyzed by this enzyme [30], [31]. The existence of a template slippage mechanism could clarify the incorporation of dAMP using DNA1 and DNA3, and of dGMP using DNA2 [29]. In any case, the most efficient dNMP incorporation was observed using DNA1 as substrate (Table 2, and Table S1).

### Influence of hXRCC1 on dNMP incorporation by DNA polymerases beta and lambda

Since hXRCC1 has been suggested to be a scaffold protein for the BER process, its effect on DNA synthesis catalyzed by DNA polymerase beta or lambda during the short-patch pathway was examined. To this end, the incorporation level of dNMP into modified duplexes (dGMP into DNA1, 3 and dAMP into DNA2, 4) was examined in the presence or absence of hXRCC1 (Fig. 2, B, C). hXRCC1 did not change the amount of product of DNA synthesis catalyzed by either DNA polymerase beta or DNA polymerase lambda.

### Strand-displacement DNA synthesis catalyzed by DNA polymerases beta and lambda

**Figure 4:**
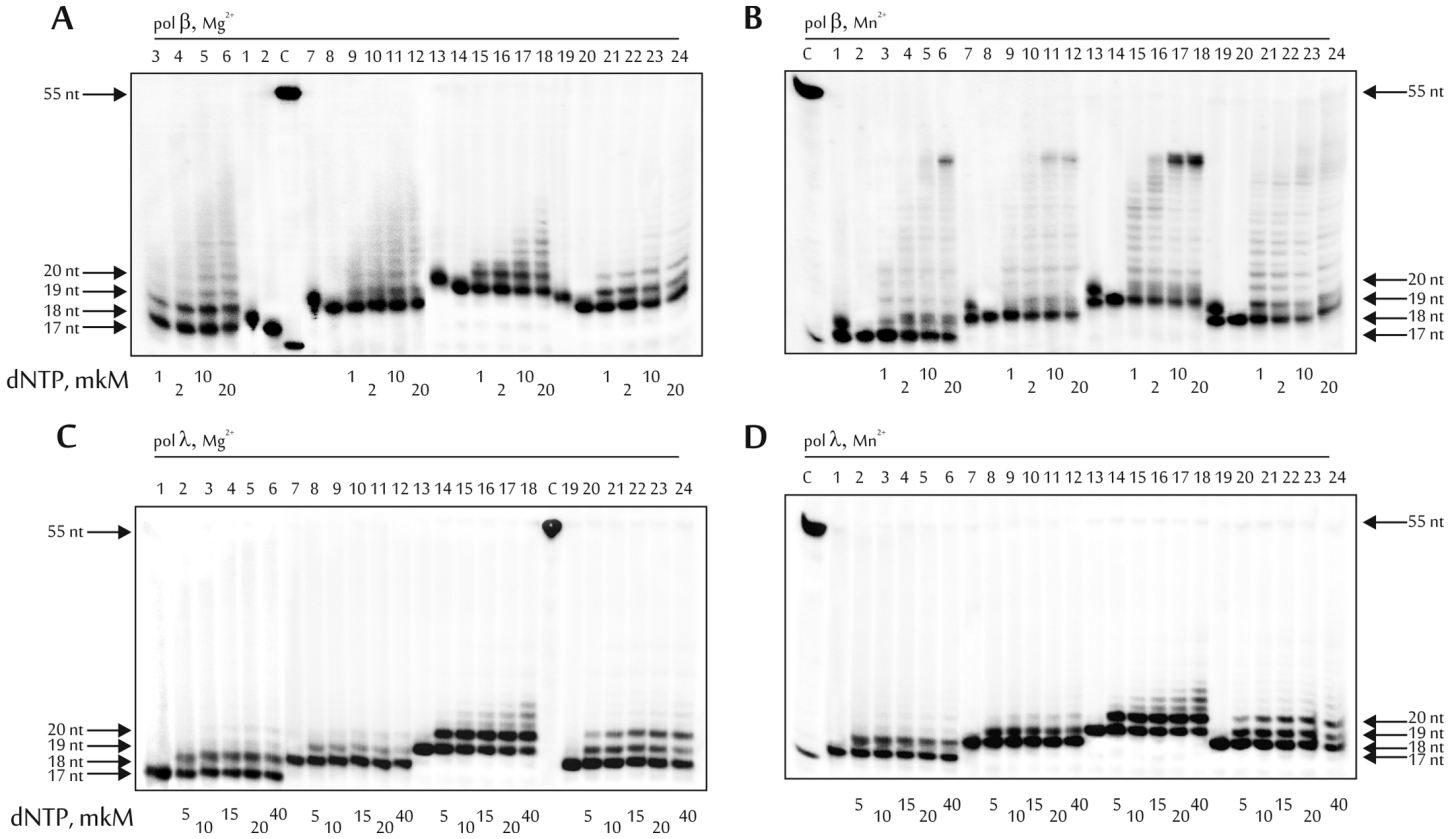
Strand-displacement activity of DNA polymerases beta (A, B) and lambda (C, D) in the presence of Mg^2+^ (A, C) or Mn^2+^ (B, D) ions using DNA1 (lanes 1–6), DNA2 (lanes 7–12), DNA3 (lanes 13–18) and DNA4 (lanes 19–24). Lane C, position of the 5'–(^32^P)labeled 55 and 18 nt-long DNA; lanes 1, 7, 13 and 19 (upper panel), initial DNA substrate after UDG treatment; lanes 2, 8, 14 and 20 (upper panel), and 1, 7, 13 and 19 (bottom panel), initial DNA substrate after sequential treatment by UDG and hAPE1; 18, 19 and 20 nt, lengths of the reaction products. The final concentrations of dNTPs are indicated below the gels. The autoradiographs present the results from one of three independent experiments.

An alternative BER pathway is the long-patch, which includes strand-displacement DNA synthesis and the generation of a flap that is removed by the flap endonuclease 1, FEN1. Upon simulation in the reconstituted system of the AP site repair within the cluster via the long-patch BER pathway, DNA synthesis catalyzed by DNA polymerase beta or lambda was carried out in the presence of all four dNTPs and hFEN1 exactly after hAPE1 cleavage (Fig. 4). In should be noted that in the absence of hFEN1, the set of products synthesized by both DNA polymerases in the presence of all four dNTPs did not differ from the products obtained under short-patch BER conditions (data not shown). DNA polymerase lambda was the less processive enzyme, and preferred a DNA substrate that contained 5-formyluridine in position −1 relative to the first incorporated nucleotide. It is possible, that interaction of the enzyme with the template 5-foU located in position +1 or +2 relative to the 3'-end of the primer is different from interaction with undamaged base. Alternatively, located in the indicated −1 position, 5-foU could change DNA flexibility and prevent normal functioning of the DNA polymerase.

The most efficient DNA synthesis catalyzed by DNA polymerase beta was also observed using DNA3 (Table 3, and Table S2). It should be noted that the strand-displacement activity of DNA polymerase beta is stimulated by hAPE1 that enables DNA polymerase beta to mediate long-patch BER pathway more effectively in comparison to DNA polymerase lambda [32].

**Table 3:** Efficiency (k_cat_/K_m_, μM^−1^⋅sec^−1^) of dNMPs incorporation by DNA polymerases beta and lambda via the long-patch BER pathway.

	pol λ		pol β	
	Mg^2+^	Mn^2+^	Mg^2+^	Mn^2+^
DNA1	0.003	0.0084	0.0084	0.012
DNA2	0.01	0.0003	0.0034	0.037
DNA3	0.022	0.0164	0.0131	0.235
DNA4	0.008	0.0072	0.0074	0.118

Note: the results are presented as the average value of three independent experiments. Standard error was estimated as 10%.

### Influence of hPCNA on the efficiency of the long-patch BER pathway

One of the inherent components of the BER system is hPCNA. First, hPCNA is a processivity factor for replicative DNA polymerases. Additionally, it participates in the coordination of protein-protein and nucleic acid-protein interactions in replication and repair complexes. Several studies have reported physical interactions between DNA polymerases beta and lambda and hPCNA [33], [34]. Evidence also exists of the influence of hPCNA on the efficiency and fidelity of translesion DNA synthesis catalyzed by DNA polymerases beta and lambda [35], [36].

**Figure 5:**
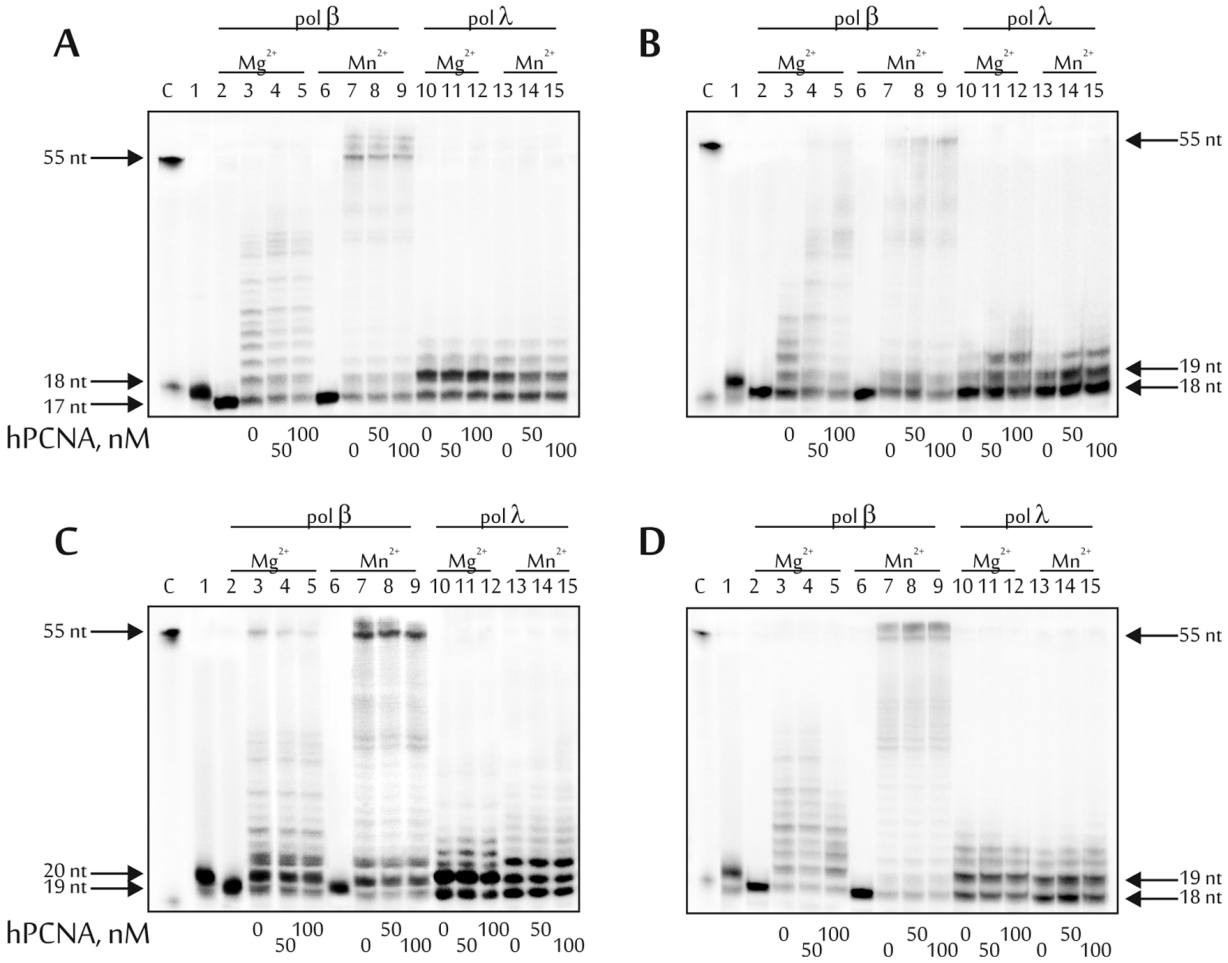
Influence of hPCNA on long-patch BER activity of DNA polymerases beta and lambda using DNA1 (A), DNA2 (B), DNA3 (C) and DNA 4 (D). Lane C, position of the 5'-(^32^P)labeled 55 and 18 nt-long DNA; lane 1, initial DNA substrate after UDG treatment; lanes 2 and 6, initial DNA substrate after sequential treatment by UDG and hAPE1; 18, 19 and 20 nt, lengths of the reaction products. The final concentrations of hPCNA are indicated below the gels. The autoradiographs present the results from one of three independent experiments.

Here, the effect of hPCNA on the ability of DNA polymerases beta and lambda to perform DNA synthesis using 5-foU-containing templates was investigated during AP site repair via the long-patch BER pathway. The effect of the protein factor was estimated by the changes in quality and amount of reaction products obtained in the presence or absence of hPCNA (Fig. 5). As can be seen from the results presented, hPCNA did not influence the yield of reaction products; however it increased DNA polymerase processivity, if the first dNMP was incorporated opposite the template 5-foU (Fig. 5B).

### AP site repair via short- and long-patch BER pathways

**Figure 6:**
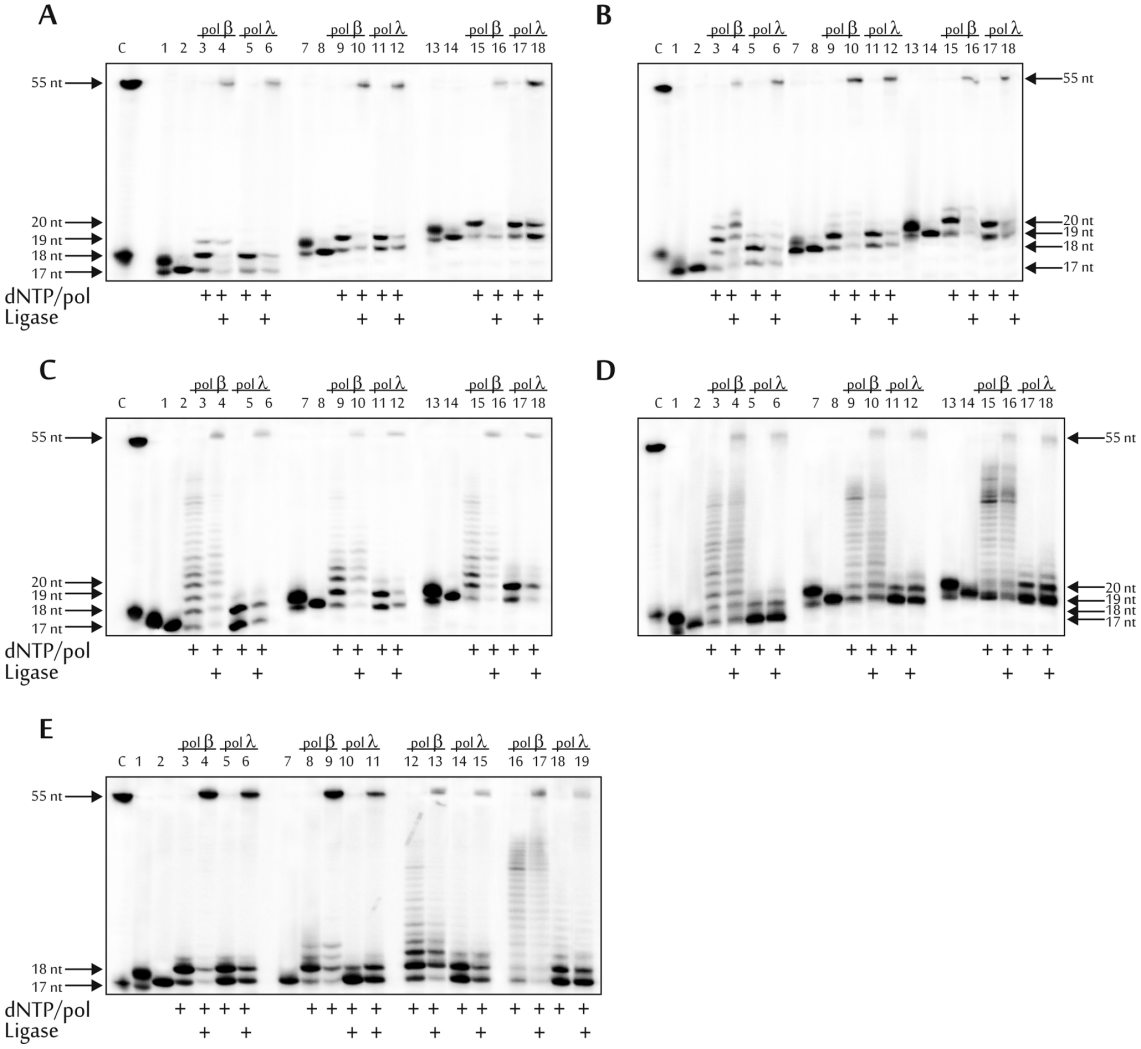
AP site repair via short-patch (A, B and E) and long-patch (C, D and E) BER pathways. Step-by-step reconstruction using DNA1(lanes 1–6 of A, B, C and D), DNA2 (lanes 7–12 of A, B, C and D), DNA3 (lanes 13–18 of A, B, C and D), and DNA4 (E). The experiments were performed in the presence of Mg^2+^ (A, C, and lanes 1–6 and 12–15 of E) or Mn^2+^ (B, D, and lanes 7–11 and 16–19 of E) ions. Lane C, position of the 5'-(^32^P)labeled 55 and 18 nt-long DNA; lanes 1, 7 and 13 (A, B, C and D) and 1 (E), initial DNA substrate after UDG treatment; lanes 2, 8 and 14 (A, B, C and D), and 2 and 7 (E), initial DNA substrate after sequential treatment with UDG and hAPE1; 18, 19, 20 and 55 nts, lengths of the reaction products. The autoradiographs present the results from one of three independent experiments.

A reconstructed system was used for repair of an AP site inside the cluster lesion. Upon modeling the short-patch BER pathway, we sequentially added hAPE1, DNA polymerase beta or lambda, and dNTP to the reaction mixture. To confirm the existence of the possible reaction product, i.e. nicked DNA with 3'-OH and 5'-phosphate groups, and to confirm its ligation, T4 DNA ligase was used (Fig. 6A, B). Reaction products whose lengths corresponded to the initial full-length DNA substrate were obtained in all cases. It should be noted that after ligation the amount of the DNA products elongated by the DNA polymerase was increased.

Similar experiments were performed to study possible repair of model DNA substrates via the long-patch BER pathway. In this case, hAPE1, DNA polymerase beta or lambda combined with hFEN1, dNTP and T4 DNA ligase were sequentially added to the reaction (Fig. 6C, D). The ligated product was obtained in all cases although in smaller amounts relative to the repair via the short-patch BER pathway. However, even in this case the products of DNA synthesis were presumably shifted to the full-length product. Additional evidence about the preferred BER pathway could be derived from analysis of the kinetics of dNTP incorporation and the influence of auxiliary factors specific for individual pathway (discussed later). It should be also noted that BER is workable via both repair pathways using a control DNA substrate containing the AP site only (Fig. 6E).

## Discussion

Radiation, radiomimetic anticancer drugs or internal active oxygen species induce high levels of clustered DNA damage, in which several individual lesions are located within one or two turns of the DNA helix. Clustered DNA damage could lead to deleterious biological consequences such as point mutations or even cell death, if the repair process becomes erroneous or incomplete. If the AP site is one of the individual lesions, it can lead to very perilous damage resulting in point mutations or single-strand breaks. In this study we analyzed step-by-step the repair of AP sites consisting of double-stranded clustered DNA damage with 5-formyl-2'-deoxyuridine which is another “hot point” for the DNA repair system. We investigated the AP-DNA repair process via the short- and long-patch BER pathways.

The capacity of the endonuclease activity of hAPE1 to cleave the AP site located in one DNA strand close to the 5-foU of the other strand was first investigated. Cleavage efficiency depends on the position of the 5-foU in the opposite DNA strand: its efficiency decreased in the row from negative to positive orientation of the lesions. Current data are in agreement with the results obtained using clustered DNA damages with the other set of individual lesions [37]. However, in terms of the next step of the BER process such hAPE1's “preferences”, these are much more severe for the cell since following DNA synthesis they could occur using damaged DNA templates. In this case, the TLS activity of the DNA polymerases beta and lambda seems promising. DNA synthesis during short-patch BER pathway catalyzed by DNA polymerase lambda was clearly more accurate than the process carried out by DNA polymerase beta. Upon reconstruction of the long-patch BER pathway, DNA polymerase beta demonstrated more processive DNA synthesis in comparison to DNA polymerase lambda. Obtained efficiencies of the dNTPs incorporation at the 3'-end of different 5-foU-contained DNA substrates lead to suggestions that the choice for DNA polymerase depends on the relative positions of 5-foU and initial AP site. Under the reaction conditions, hPCNA, which normally acts as co-factor of long-patch BER pathway, stimulates strand-displacement activity of both enzymes using DNA2 as substrate, in which the 5-foU is located opposite the first incorporated nucleotide. It should be noted that no effect of hXRCC1 on DNA polymerase and hAPE1 activities was detected. These observations were probably due to the incomplete reaction conditions required for protein-protein interaction within the complicated BER machine. Since during the process XRCC1 is strongly associated with Ligase III [18], the lack of hXRCC1 effect might be explained by the absence of this enzyme.

**Figure 7:**
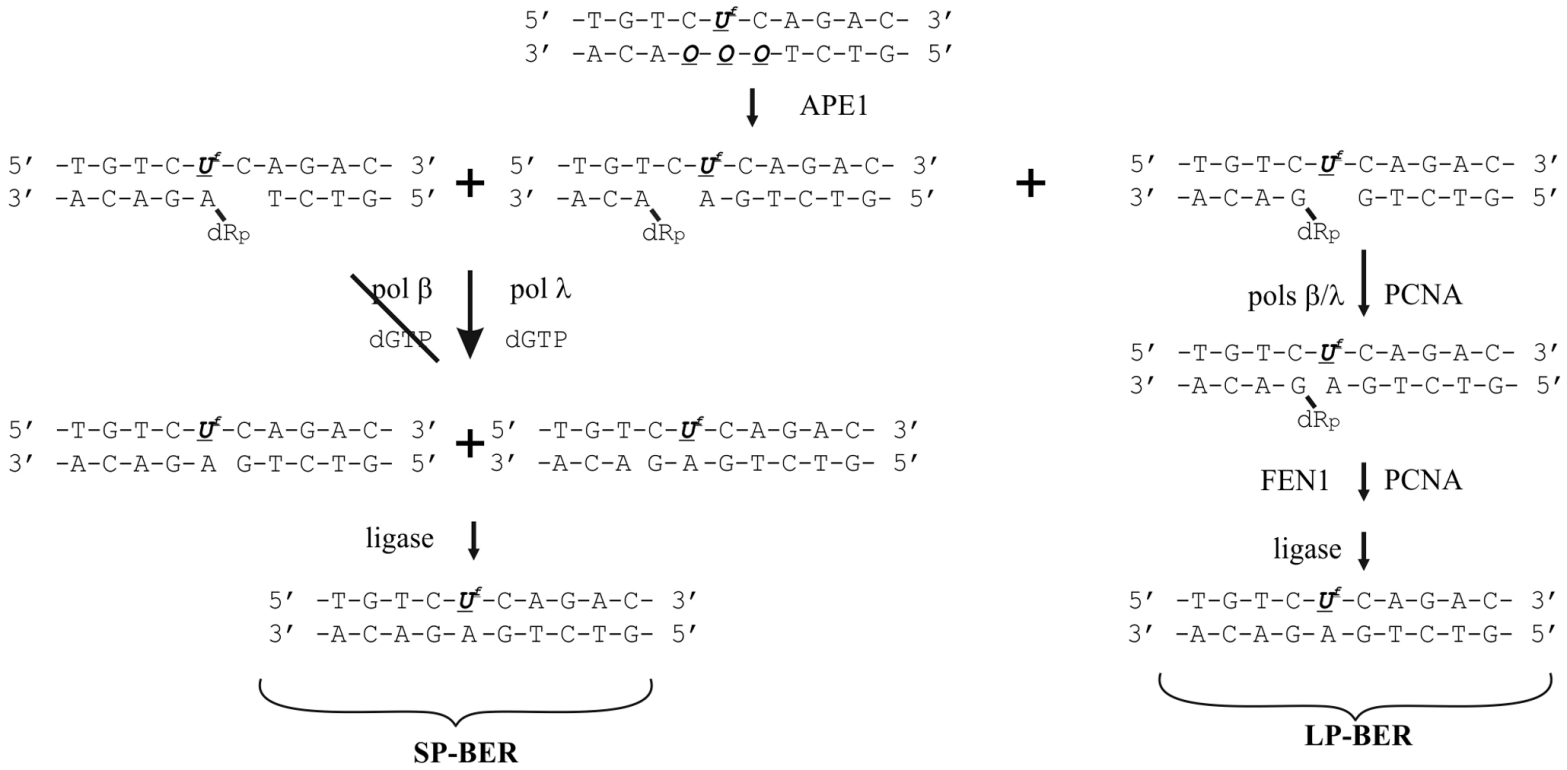
BER pathways for repair of AP site introduced into the cluster damage with 5-foU in the opposite strand. ***U^f^***, 5-foU, ***O***, AP site.

Thus, based on the results obtained we propose the following summary (Fig. 7). The AP site being part of the cluster damage with 5-foU can be repaired through the BER process. If the AP site is shifted relative to the 5-foU of the opposite strand it could be repaired primarily via the short-patch BER pathway. In this case, DNA synthesis catalyzed by DNA polymerase lambda could be more accurate, and, therefore, point mutations would not accumulate. If the AP site is located exactly opposite 5-foU it is more likely to switch the repair to the long-patch BER pathway. In this situation, hPCNA stimulates the strand-displacement activity of both DNA polymerases.

## Supporting Information

Figure S1 **Complex formation between recombinant hXRCC1 and either hAPE1 (A) or DNA polymerase beta (B).** Initially, hXRCC1 was incubated with hAPE1 or DNA polymerase beta, then Ni-NTA agarose (®Qiagen) was added to the mixture. After incubation Ni-NTA agarose was gently pelleted, the supernatant containing non-adsorbed material was removed (lanes indicated as FT, flow-through sample), and the Ni-NTA agarose beads washed several times with buffesr containing 25 mM imidazole (lanes indicated as 25). Proteins were finally eluted from the Ni-NTA agarose beads by washing several times with buffer containing 250 mM imidazole (lanes indicated as 250). All samples were examined by Coomassie blue R250 staining of SDS-PAGE. Lanes M – molecular weight markers.
(TIF)

Table S1 **Kinetic parameters (K_m_, V_max_) of dNMP incorporation by DNA polymerases beta and lambda via the short-patch BER pathway.**
(DOC)

Table S2 **Kinetic parameters (K_m_, V_max_) of dNMPs incorporation by DNA polymerases beta and lambda via the long-patch BER pathway.**
(DOC)

## References

[1] Semenenko VA, Stewart RD (2004) A fast Monte Carlo algorithm to simulate the spectrum of DNA damages formed by ionizing radiation. Radiat Res 161: 451–457. 15038766 10.1667/rr3140

[2] Adelman R, Saul RL, Ames BN (1988) Oxidative damage to DNA: relation to species metabolic rate and life span. Proc Natl Acad Sci U S A. 85: 2706–2708. 10.1073/pnas.85.8.2706 PMC280067 3128794

[3] Bjelland S, Anensen H, Knaevelsrud I, Seeberg E (2001) Cellular effects of 5-formyluracil in DNA. Mutat Res 486: 147–154. 11425519 10.1016/s0921-8777(01)00085-4

[4] Kamiya H, Murata-Kamiya N, Karino N, Ueno Y, Matsuda A, et al (2002) Induction of T –> G and T –> A transversions by 5-formyluracil in mammalian cells. Mutat Res 513: 213–222. 11719107 10.1016/s1383-5718(01)00312-6

[5] Dohno C, Okamoto A, Saito I (2005) Stable, specific, and reversible base pairing via Schiff base. J Am Chem Soc 127: 16681–16684. 16305258 10.1021/ja054618q

[6] Bjelland S, Eide L, Time RW, Stote R, Eftedal I, et al (1995) Oxidation of thymine to 5-formyluracil in DNA: mechanisms of formation, structural implications, and base excision by human cell free extracts. Biochemistry 34: 14758–14764. 7578084 10.1021/bi00045a017

[7] de Los Santos C, El-Khateeb M, Rege P, Tian K, Johnson F (2004) Impact of the C1' configuration of abasic sites on DNA duplex structure. Biochemistry 43: 15349–15357. 15581347 10.1021/bi048400c

[8] Guerniou V, Rapin D, Millau JF, Bufflier E, Favier A, et al (2005) Repair of oxidative damage of thymine by HeLa whole-cell extracts: simultaneous analysis using a microsupport and comparison with traditional PAGE analysis. Biochimie 87: 151–159. 15760707 10.1016/j.biochi.2004.12.001

[9] Eot-Houllier G, Eon-Marchais S, Gasparutto D, Sage E (2005) Processing of a complex multiply damaged DNA site by human cell extracts and purified repair proteins. Nucleic Acids Res 33: 260–271. 15647508 10.1093/nar/gki165 PMC546153

[10] Zhang QM (2001) Role of the Escherichia coli and Human DNA Glycosylases That Remove 5-Formyluracil from DNA in the Prevention of Mutations. J Radiat Res 42: 11–19. 11393886 10.1269/jrr.42.11

[11] Knævelsrud I, Slupphaug G, Leiros I, Matsuda A, Ruoff P, et al (2009) Opposite-base dependent excision of 5-formyluracil from DNA by hSMUG1. Int J Radiat Biol 85: 413–420. 19365746 10.1080/09553000902818915

[12] Kino K, Shimizu Y, Sugasawa K, Sugiyama H, Hanaoka F (2004) Nucleotide Excision Repair of 5-Formyluracil in Vitro Is Enhanced by the Presence of Mismatched Bases. Biochemistry 43: 2682–2687. 15005603 10.1021/bi0361416

[13] Irie D, Ono A, Izuta S (2006) Recognition of oxidized thymine base on the single-stranded DNA by replication protein A. Nucleosides, Nucleotides, and Nucleic Acids. 25: 439–451. 10.1080/01457630600684138 16838837

[14] Belousova E, Lavrik O (2010) DNA Polymerases beta and lambda and Their Roles in DNA Replication and Repair. Mol Biol (Mosk) 44: 839–855. 21290819

[15] Braithwaite E, Prasad R, Shock D, Hou E, Beard W, et al (2005) DNA polymerase lambda mediates a back-up base excision repair activity in extracts of mouse embryonic fibroblasts. J Biol Chem 280: 18469–1847. 15749700 10.1074/jbc.M411864200

[16] Date T, Yamaguchi M, Hirose F, Nishimoto Y, Tanihara K, et al (1998) Expression of active rat DNA polymerase beta in Escherichia coli. Biochemistry 27: 2983–2990. 10.1021/bi00408a048 3042024

[17] Jonsson Z, Hindges R, Hubscher U (1998) Regulation of DNA replication and repair proteins through interaction with the front side of proliferating cell nuclear antigen. EMBO J 17: 2412–2425. 9545252 10.1093/emboj/17.8.2412 PMC1170584

[18] Caldecott K, Tucker J, Stanker L, Thompson L (1995) Characterization of the XRCC1-DNA ligase III complex in vitro and its absence from mutant hamster cells. Nucleic Acids Res 23: 4836–4643. 8532526 10.1093/nar/23.23.4836 PMC307472

[19] Zatsepin TS, Stetsenko DA, Arzumanov AA, Romanova EA, Gait MJ, et al (2002) Synthesis of peptide−oligonucleotide conjugates with single and multiple peptides attached to 2’-aldehydes through thiazolidine, oxime, and hydrazine linkages. Bioconjugate Chem 13: 822–830. 10.1021/bc020016+ 12121138

[20] Mazin A (1990) Label's introducing into DNA in Methods of molecular genetics and gene engineering. In: Salganik R, editor. Novosibirsk: Science SBRAS. 25–26.

[21] Sambrook J, Fritsch E, Maniatis T (1989) Molecular Cloning: A Laboratory Manual, Cold Spring Harbor Laboratory Press. Cold Spring Harbor. New York.

[22] Leskovac V (2004) Comprehensive Enzyme Kinetics. In: Kluwer Academic Publishers, editor. New York, Boston, Dordrecht, London, Moscow. 31–49.

[23] Dianov G, Sleeth K, Dianova I, Allinson S (2003) Repair of abasic sites in DNA. Mutat Res 531: 157–163. 14637252 10.1016/j.mrfmmm.2003.09.003

[24] Tsunoda M, Sakaue T, Naito S, Sunami T, Abe N, et al.. (2010) Insights into the structures of DNA damaged by hydroxyl radical: crystal structures of DNA duplexes containing 5-formyluracil. J Nucleic Acids doi: 10.4061/2010/107289. 10.4061/2010/107289 PMC2952808 20976303

[25] Oezguen N, Schein CH, Peddi SR, Power TD, Izumi T, et al (2007) A “moving metal mechanism” for substrate cleavage by the DNA repair endonuclease APE-1. Proteins 68: 313–323. 17427952 10.1002/prot.21397

[26] Vidal A, Boiteux S, Hickson I, Radicella J (2001) XRCC1 coordinates the initial and late stages of DNA abasic site repair through protein–protein interactions. EMBO J 20: 6530–6539. 11707423 10.1093/emboj/20.22.6530 PMC125722

[27] Kubota Y, Nash RA, Klungland A, Schär P, Barnes D, et al (1996) Reconstitution of DNA base excision-repair with purified human proteins: interaction between DNA polymerase beta and the XRCC1 protein. EMBO J 15: 6662–6670. 8978692 PMC452490

[28] Volk D, Thiviyanathan V, Somasunderam A, Gorenstein D (2007) Ab initio base-pairing energies of an oxidized thymine product, 5-formyluracil, with standard DNA bases at the BSSE-free DFT and MP2 theory levels. Org Biomol Chem 5: 1554–1558. 17571183 10.1039/b702755a

[29] Blanca G, Villani G, Shevelev I, Ramadan K, Spadari S, et al (2004) Human DNA polymerases lambda and beta show different efficiencies of translesion DNA synthesis past abasic sites and alternative mechanisms for frameshift generation. Biochemistry 43: 11605–11615. 15350147 10.1021/bi049050x

[30] Kunkel TA, Alexander SJ (1986) The base substitution fidelity of eukaryotic DNA polymerases. Mispairing frequencies, site preferences, insertion preferences, and base substitution by dislocation. J Biol Chem 261: 160–166. 3941068

[31] Lin P, Batra VK, Pedersen LC, Beard WA, Wilson SH, et al (2008) Incorrect nucleotide insertion at the active site of a G:A mismatch catalyzed by DNA polymerase beta. Proc Natl Acad Sci U S A 105: 5670–5674. 18391201 10.1073/pnas.0801257105 PMC2311328

[32] Sukhanova M, Khodyreva S, Lebedeva N, Prasad R, Wilson S, et al (2005) Human base excision repair enzymes apurinic/apyrimidinic endonuclease1 (APE1), DNA polymerase beta and poly(ADP-ribose) polymerase 1: interplay between strand-displacement DNA synthesis and proofreading exonuclease activity. Nucleic Acids Res 33: 1222–1229. 15731342 10.1093/nar/gki266 PMC549570

[33] Kedar P, Kim S, Robertson A, Hou E, Prasad R, et al (2002) Direct interaction between mammalian DNA polymerase beta and proliferating cell nuclear antigen. J Biol Chem 277: 31115–311123. 12063248 10.1074/jbc.M201497200

[34] Shimazaki N, Yazaki T, Kubota T, Sato A, Nakamura A, et al (2005) DNA polymerase lambda directly binds to proliferating cell nuclear antigen through its confined C-terminal region. Genes Cells 10: 705–715. 15966901 10.1111/j.1365-2443.2005.00868.x

[35] Maga G, Villani G, Crespan E, Wimmer U, Ferrari E, et al (2007) 8-oxo-guanine bypass by human DNA polymerases in the presence of auxiliary proteins. Nature 447: 606–608. 17507928 10.1038/nature05843

[36] Belousova E, Maga G, Fan Y, Kubareva E, Romanova E, et al (2010) DNA polymerases beta and lambda bypass thymine glycol in gapped DNA structures. Biochemistry 49: 4695–4704. 20423048 10.1021/bi901792c

[37] Paap B, Wilson DM III, Sutherland BM (2008) Human abasic endonuclease action on multilesion abasic clusters: implications for radiation-induced biological damage. Sutherland Nucleic Acids Res 36: 2717–2727. 18353858 10.1093/nar/gkn118 PMC2377450

