# Supplementary figures and images for: Clustered DNA Lesions Containing 5-Formyluracil and AP Site: Repair via the BER System
Source: PLoS One. 2013 Aug 6;8(8):e68576. doi: 10.1371/journal.pone.0068576 (PMC3735541; doi:10.1371/journal.pone.0068576)

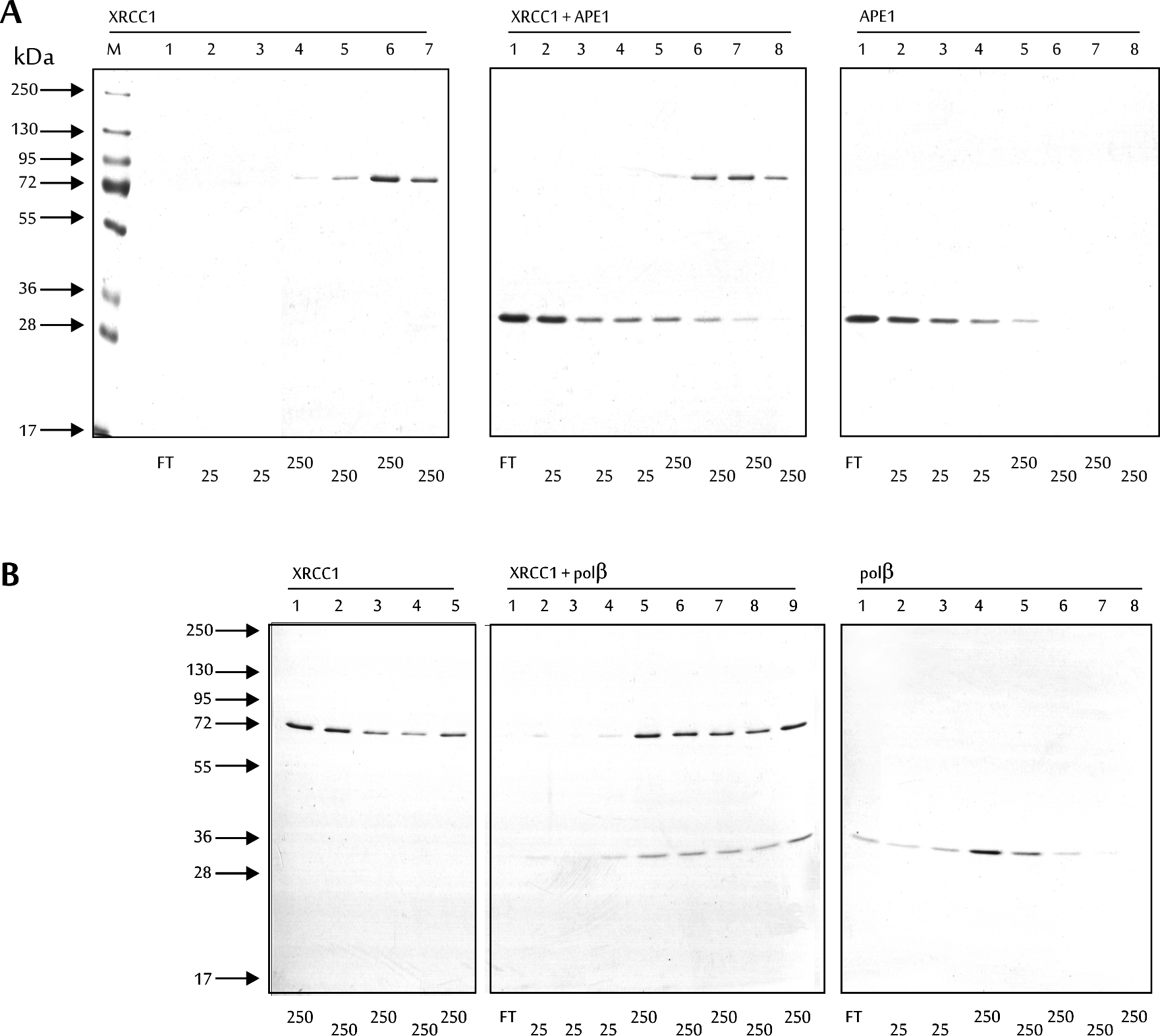

Supplement: Figure S1 — Complex formation between recombinant hXRCC1 and either hAPE1 (A) or DNA polymerase beta (B). Initially, hXRCC1 was incubated with hAPE1 or DNA polymerase beta, then Ni-NTA agarose (®Qiagen) was added to the mixture. After incubation Ni-NTA agarose was gently pelleted, the supernatant containing non-adsorbed material was removed (lanes indicated as FT, flow-through sample), and the Ni-NTA agarose beads washed several times with buffesr containing 25 mM imidazole (lanes indicated as 25). Proteins were finally eluted from the Ni-NTA agarose beads by washing several times with buffer containing 250 mM imidazole (lanes indicated as 250). All samples were examined by Coomassie blue R250 staining of SDS-PAGE. Lanes M – molecular weight markers. (TIF) [file pone.0068576.s001.tif]
