# Supplementary material for: Clustered DNA Lesions Containing 5-Formyluracil and AP Site: Repair via the BER System
Source: PLoS One. 2013 Aug 6;8(8):e68576. doi: 10.1371/journal.pone.0068576 (PMC3735541; doi:10.1371/journal.pone.0068576)
Supplement: Table S1 — Kinetic parameters (Km, Vmax) of dNMP incorporation by DNA polymerases beta and lambda via the short-patch BER pathway. (DOC) [file pone.0068576.s002.doc]

**Table S1.** Kinetic parameters (K_m_, V_max_) of dNMP incorporation by DNA polymerases beta and lambda via the short-patch BER pathway.

|  | V_max_, nM⋅sec^-1^ | K_m_, nM | k_cat_, sec^-1^ | k_cat_/K_m_, μM^-1^⋅sec^-1^ | V_max_, nM⋅sec^-1^ | K_m_, nM | k_cat_, sec^-1^ | k_cat_/K_m_, μM^-1^⋅sec^-1^ |
| --- | --- | --- | --- | --- | --- | --- | --- | --- |
|  | Mg^2+^ | | | | Mn^2+^ | | | |
|  | pol λ | | | | | | | |
| DNA1/dGTP | 0.53 | 311 | 0.05 | 0.171 | 1.35 | 37.2 | 0.14 | 3.62 |
| DNA2/dATP | 0.15 | 428 | 0.015 | 0.036 | 0.37 | 85.3 | 0.04 | 0.44 |
| DNA3/dGTP | 0.22 | 1101 | 0.02 | 0.020 | 1.10 | 38.9 | 0.11 | 2.3 |
| DNA4/dATP | 0.22 | 3749 | 0.02 | 0.006 | 0.40 | 175 | 0.04 | 0.23 |
|  | pol β | | | | | | | |
| DNA1/dGMP | 3.05 | 77.8 | 0.28 | 3.92 | 4.20 | 12.9 | 0.42 | 32.6 |
| DNA2/dAMP | 1.52 | 188 | 0.12 | 0.81 | 1.87 | 22.8 | 0.19 | 8.20 |
| DNA3/dGMP | 2.04 | 86.7 | 0.12 | 2.36 | 1.81 | 19.3 | 0.18 | 9.35 |
| DNA4/dAMP | 2.75 | 240 | 0.34 | 1.14 | 5.04 | 5.25 | 0.50 | 96.0 |

Note: the results are presented as the average value of three independent experiments. Standard error was estimated as 10%.
