# Supplementary material for: Clustered DNA Lesions Containing 5-Formyluracil and AP Site: Repair via the BER System
Source: PLoS One. 2013 Aug 6;8(8):e68576. doi: 10.1371/journal.pone.0068576 (PMC3735541; doi:10.1371/journal.pone.0068576)
Supplement: Table S2 — Kinetic parameters (Km, Vmax) of dNMPs incorporation by DNA polymerases beta and lambda via the long-patch BER pathway. (DOC) [file pone.0068576.s003.doc]

**Table S2.** Kinetic parameters (K_m_, V_max_) of dNMPs incorporation by DNA polymerases beta and lambda via the long-patch BER pathway.

|  | V_max_, nM⋅sec^-1^ | K_m_, nM | k_cat_, sec^-1^ | k_cat_/K_m_, μM^-1^⋅sec^-1^ | V_max_, nM⋅sec^-1^ | | K_m_, nM | k_cat_, sec^-1^ | k_cat_/K_m_, μM^-1^⋅sec^-1^ |
| --- | --- | --- | --- | --- | --- | --- | --- | --- | --- |
|  | Mg^2+^ | | | | Mn^2+^ | | | | |
|  | pol λ | | | | | | | | |
| DNA1 | 0.26 | 9.13 | 0.026 | 0.003 | 0.20 | 2.40 | | 0.020 | 0.0084 |
| DNA2 | 0.25 | 2.73 | 0.026 | 0.01 | 0.11 | 37.6 | | 0.011 | 0.0003 |
| DNA3 | 0.81 | 3.64 | 0.081 | 0.022 | 0.87 | 5.33 | | 0.087 | 0.0164 |
| DNA4 | 0.82 | 10.2 | 0.082 | 0.008 | 0.62 | 8.59 | | 0.062 | 0.0072 |
|  | pol β | | | | | | | | |
| DNA1 | 0.34 | 4.04 | 0.034 | 0.0084 | 0.34 | 2.74 | | 0.034 | 0.012 |
| DNA2 | 0.45 | 13.4 | 0.045 | 0.0034 | 0.28 | 0.75 | | 0.028 | 0.037 |
| DNA3 | 0.30 | 2.27 | 0.030 | 0.0131 | 0.41 | 0.18 | | 0.041 | 0.235 |
| DNA4 | 0.26 | 3.57 | 0.026 | 0.0074 | 0.41 | 0.34 | | 0.041 | 0.118 |

Note: the results are presented as the average value of three independent experiments. Standard error was estimated as 10%.
